# Cell-Free RNA Content in Peripheral Blood as Potential Biomarkers for Detecting Circulating Tumor Cells in Non-Small Cell Lung Carcinoma

**DOI:** 10.3390/ijms17111845

**Published:** 2016-11-05

**Authors:** Xin-Min Yu, Yi-Chen Wu, Xiang Liu, Xian-Cong Huang, Xiu-Xiu Hou, Jiu-Li Wang, Xiang-Liu Cheng, Wei-Min Mao, Zhi-Qiang Ling

**Affiliations:** 1 Zhejiang Cancer Research Institute, Zhejiang Province Cancer Hospital, Zhejiang Cancer Center, Hangzhou 310022, China; yuxinmin19750705@outlook.com (X.-M.Y.); wuyichen19870807@hotmail.com (Y.-C.W.); liuxiang19880808@outlook.com (X.L.); huangxiancong@hotmail.com (X.-C.H.); houxiuxiu@hotmail.com (X.-X.H.); wangjl1988@outlook.com (J.-L.W.); chen_xiangliu@outlook.com (X.-L.C.); maowm88@outlook.com (W.-M.M.); 2 Department of Thoracic Tumor Chemotherapy, Zhejiang Province Cancer Hospital, Zhejiang Cancer Center, Hangzhou 310022, China; 3 Zhejiang Key Laboratory of Diagnosis & Treatment Technology on Thoracic Oncology (Lung and Esophagus), Hangzhou 310022, China

**Keywords:** non-small-cell lung carcinoma (NSCLC), circulating tumor cells (CTCs), peripheral blood mononuclear cells (PBMCs), cell-free RNA content, prognosis

## Abstract

Circulating tumor cells (CTCs) have been implicated in tumor progression and prognosis. Techniques detecting CTCs in the peripheral blood of patients with non-small cell lung carcinoma (NSCLC) may help to identify individuals likely to benefit from early systemic treatment. However, the detection of CTCs with a single marker is challenging, owing to low specificity and sensitivity and due to the heterogeneity and rareness of CTCs. Herein, the probability of cell-free RNA content in the peripheral blood as a potential biomarker for detecting CTCs in cancer patients was investigated. An immunomagnetic enrichment of real-time reverse-transcription PCR (RT-PCR) technology for analysis of CTCs in NSCLC patients was also developed. The mRNA levels of four candidate genes, cytokeratin 7 (*CK7*), E74-like factor 3 (*ELF3*), epidermal growth factor receptor (*EGFR*), and erythropoietin-producing hepatocellular carcinoma receptor B4 (*EphB4*) that were significantly elevated in tumor tissues and peripheral blood mononuclear cells (PBMCs) were determined. The expression of *CK7* and *ELF3* in tumor tissues and *EGFR* in PBMCs was associated with lymph node metastasis (all *p* < 0.05). The expression of *CK7* in PBMCs was correlated with age and *EphB4* in PBMCs correlated with histopathological type, respectively (all *p* < 0.05). The expression of all four genes in tumor tissues and PBMCs was significantly correlated with the clinical stage (all *p* < 0.01). Survival analysis showed that the patients with enhanced expression of *CK7*, *ELF3*, *EGFR*, and *EphB4* mRNA in PBMCs had poorer disease-free survival (DFS) and overall survival (OS) than those without (all *p* < 0.0001). The present study showed that this alteration of cell-free RNA content in peripheral blood might have clinical ramifications in the diagnosis and treatment of NSCLC patients.

## 1. Introduction

The continuous search for tumor markers has yielded several positive results [1]. Research on biomarkers playing a vital role in the early diagnosis and individualized treatments of the tumors is rapidly advancing as we begin to understand the complex mechanisms during tumor carcinogenesis and progression [1]. The identification of molecular subtypes of non-small cell lung carcinoma (NSCLC) has transformed the clinical management of this disease, which is best exemplified by the clinical success of targeting the epidermal growth factor receptor (EGFR) or anaplastic lymphoma kinase (ALK) with tyrosine kinase inhibitors (TKI) as the first line of treatment [2]. However, lung cancer is still the leading cause of cancer mortality worldwide. The high mortality is due to the poor prognosis of the disease caused by late presentation of illness, tumor heterogeneities within histological subtypes, and the limited understanding of tumor biology. Importantly, difficulty in early diagnosis of lung cancer is due to the lack of a quintessential biomarker [3]. Recently, invasive biomarkers present in sputum, plasma, serum, or whole blood have increasingly been explored for the early diagnosis of NSCLC.

The practice of liquid biopsy as a diagnostic, prognostic, and theranostic tool in NSCLC patients is an appealing approach since it is noninvasive and easily reproducible. In particular, this method for the potential detection of circulating biomarkers allows patient monitoring during treatment, as well as the detection of different genomic alterations that may be accessible to targeted therapy or are associated with treatment resistance [4]. Recently, detection and molecular characterization of circulating tumor cells (CTCs) are some of the most active areas in translational cancer research; the use of CTCs as a liquid biopsy may aid in obtaining genetic follow-up data, which is an urgent prerequisite [5]. However, the challenge in the detection of CTCs is the requirement of high sensitivity combined with high specificity [5]. The high heterogeneity and low occurrence frequencies of CTCs which have been detected for a low concentration of 1–10 CTCs/mL in whole blood of patients with metastasis, hinder the development of clinical applications [4,6].

Methods for CTC detection have recently been developed, including CTC microchips, filtration devices, quantitative reverse-transcription PCR assays, and automated microscopy systems [4,7]. A successful detection of rare CTCs in peripheral blood can be achieved by coupling pre-analytical enrichment with molecular detection of enriched cells by reverse-transcription-PCR (RT-PCR) [8]. CTC detection by a panel of markers that show increased expression in tumor cells as compared to the normal epithelial cells is a critical supplement to the current tumor-node-metastasis (TNM) staging system for improved prognosis and rapid assessment of the therapeutic response. These improvements facilitate the design of enhanced therapeutic strategies for the treatment of any solid epithelial cancer [9,10]. Although many promising CTC detection techniques have been developed in recent years, the analytical specificity and specificity of these methods require substantiation in large prospective multicenter studies before clinical utility. In addition, the cost-efficiency of the detection method should also be considered.

Herein, immunobead RT-PCR was developed for the detection of CTCs originating from NSCLC. The technique combined pre-analytical enrichment by density gradient centrifugation (Ficoll-Hypaque separation), immunomagnetic, and size filtration procedures with subsequent RT-PCR detection of a panel of four markers. The markers used for RT-PCR included *CK7*, *ELF3*, *EGFR*, and *EphB4*, which were selected based on the expression in epithelial cancers including NSCLC, as described previously [11,12,13,14]. These markers can be used for the identification of circulating breast cancer cells [12], and cannot be detected in mononuclear cells from peripheral blood samples in normal individuals [12]. Therefore, these markers are specific for the detection of circulating epithelial cells.

## 2. Results

### 2.1. Expression Level of CK7, ELF3, EGFR, and EphB4 mRNA in NSCLC Tissues

**Figure 1:**
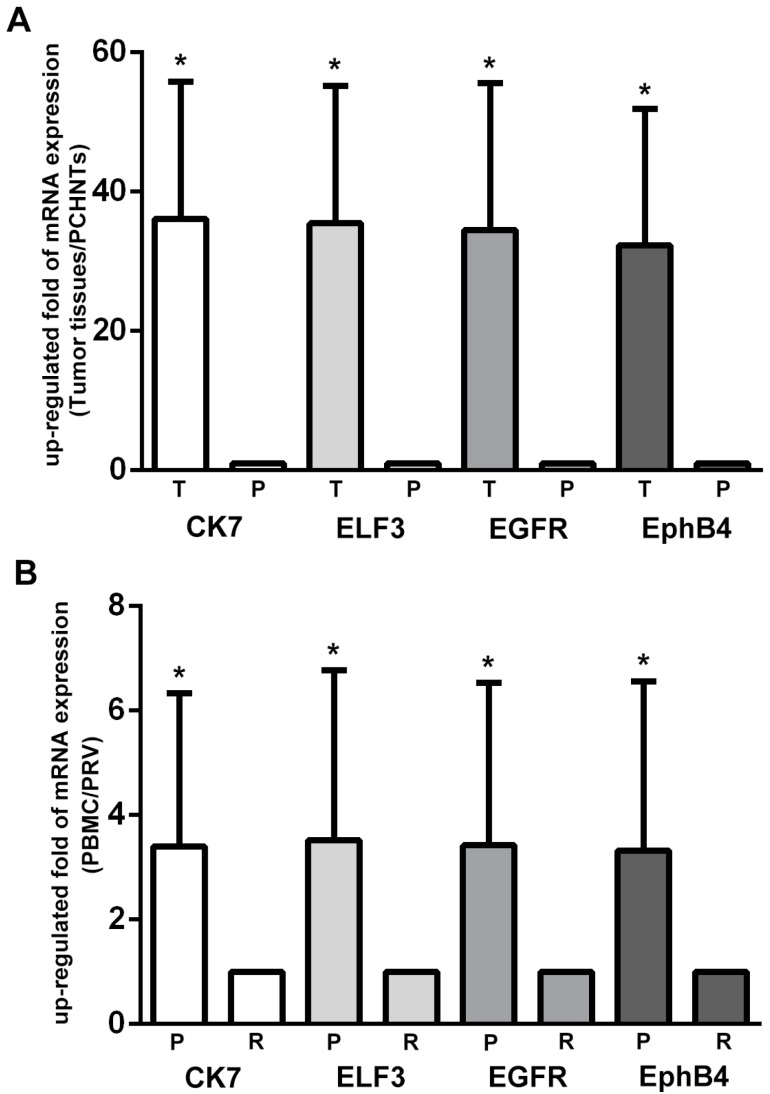
Real-time reverse-transcription PCR (RT-PCR) analysis of marker gene expression in non-small cell lung carcinoma (NSCLC) tissues and paired peripheral blood mononuclear cell (PBMC) specimens. Total amount of mRNA was normalized to *GAPDH* and the relative mRNA expressions of the samples (tumor vs. para-cancerous histological normal tissues (PCHNTs), or paired PBMCs vs. positive control) were calculated using 2^−ΔΔ*C*t^ formula. (**A**) Tumor tissues vs. PCHNTs from 111 NSCLC patients. The mRNA expression of *CK7*, *ELF3*, *EGFR* and *EphB4* in lung cancer tissues were significantly higher than that in PCHNTs (all *p* < 0.0001); T: Tumor tissues; P: PCHNTs; (**B**) PBMCs from 111 NSCLC patients vs. positive reference value (PRV). The mRNA expression of *CK7*, *ELF3*, *EGFR* and *EphB4* in PBMCs from NSCLC patients were significantly higher than that in healthy controls, respectively (all *p* < 0.0001); P: PBMC; R: PRV. *: *p* ˂ 0.0001.

The mRNA level of *CK7*, *ELF3*, *EGFR*, and *EphB4* in NSCLC tissues was significantly higher than that in para-cancerous histological normal tissues (PCHNTs) (all *p* < 0.0001, respectively, Figure 1A). The average increase of mRNA of *CK7*, *ELF3*, *EGFR*, and *EphB4* was 36.118-, 35.476-, 34.541-, and 32.308-fold, respectively. Successively, the correlation between the four mRNA levels and the clinicopathological characteristics was analyzed (Table 1). The results indicated that the expression level of the four mRNAs was closely correlated with the clinical stage, respectively (all *p* < 0.0001). However, no correlation could be established between the levels of the four mRNAs and age, sex, and smoking history, respectively (all *p* > 0.05, Table 1).

### 2.2. The mRNA Expression of Selected Markers in Paired PBMC Preparations

346 blood samples were taken from the recruited participants including 111 NSCLC patients, 115 benign pulmonary disease patients, and 120 healthy controls. No significant difference was observed in the age and gender among the different groups *(p* > 0.05 for both).

The expression of the four putative markers identified in paired PBMCs was validated by real-time RT-PCR. We found that these four mRNAs were detectable in the majority of PBMC samples from 111 NSCLC patients. Four markers (*CK7*, *ELF3*, *EGFR*, and *EphB4*) were significantly elevated in PBMCs (all *p* ˂ 0.0001) (Figure 1B). However, no significant difference was observed in the expression of the four genes in PBMCs from 115 benign pulmonary disease patients as compared to 120 healthy controls (*p* > 0.05). Furthermore, the results indicated that these four mRNA levels in PBMCs was closely correlated with the clinical stage (*p* = 0.006, 0.003, 9.62 × 10^−5^, and 0.006, respectively) in NSCLC patients (Table 2). The circulating *CK7* mRNA was closely correlated with the patient’s age revealed during the first examination (*p* = 0.038). The circulating *EphB4* mRNA was closely correlated with the histopathological type (*p* = 0.032) while the circulating *EGFR* mRNA was closely correlated with the lymph node metastasis (*p* = 0.023) (Table 2).

### 2.3. Receiver Operating Characteristics (ROC) Curve Analysis of the Four mRNAs as a Marker of CTCs

**Figure 2:**
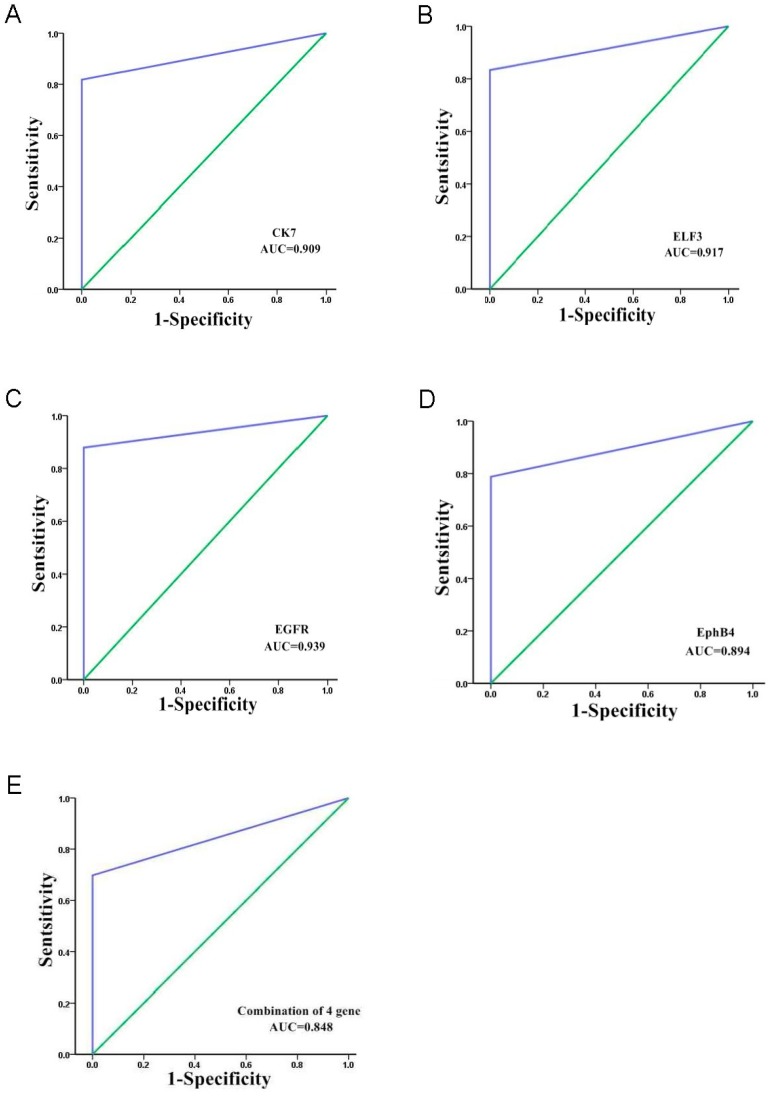
Receiver operating characteristics (ROC) curve analysis of the four mRNAs as a marker of circulating tumor cells (CTCs). The areas under the ROC curves are as follows: (**A**) *CK7*, 0.909 (0.853–0.965, 95.0% CI) with a sensitivity of 81.8% and a specificity of 100.0% for the discrimination between CTCs and the normal subjects; (**B**) *ELF3*, 0.917 (0.863–0.971, 95.0% CI) with a sensitivity of 83.3% and a specificity of 100.0% for the discrimination between CTCs and the normal subjects; (**C**) *EGFR*, 0.939 (0.893–0.986, 95.0% CI) with a sensitivity of 87.8% and a specificity of 100.0% for the discrimination between CTCs and the normal subjects; (**D**) *EphB4*, 0.894 (0.834–0.954, 95.0% CI) with a sensitivity of 78.8% and a specificity of 100.0% for the discrimination between CTCs and the normal subjects; (**E**) The area under the ROC curve was 0.848 (0.779–0.917, 95.0% CI), the sensitivity and specificity were 69.7% and 100%, respectively, when CTCs was detected using the combination of four markers; AUC: Area under curve.

The areas under the ROC curves were 0.909 (0.853–0.965, 95.0% confidence interval (CI)) for *CK7*, 0.917 (0.863–0.971, 95.0% CI) for *ELF3*, 0.939 (0.893–0.986, 95.0% CI) for *EGFR*, and 0.894 (0.834–0.954, 95.0% CI) for *EphB4* (Figure 2). The cut-off values of *CK7*, *ELF3*, *EGFR*, and *EphB4* were defined as a 2-fold increase of the expression compared with the positive control. The sensitivity of each marker was: *CK7* (81.8%), *ELF3* (83.3%), *EGFR* (87.8%), *EphB4* (78.8%), whereas the specificity was: *CK7* (100%), *ELF3* (100%), *EGFR* (100%), and *EphB4* (100%). The expression of at least one of these four markers (a combination of the four markers) was considered as CTC positive. Thus, we determined the effect of the combination of the four markers by constructing a ROC curve and fitting a logistic model with parameters for *CK7*, *ELF3*, *EGFR* and, *EphB4*. The area under the ROC curve was 0.848 (0.779–0.917, 95.0% CI); the sensitivity and specificity were 69.7% and 100%, respectively, when the optimal threshold was defined as a 2-fold increase of expression (Figure 2). These results indicated that the detection of CTCs using the combination of four markers was not superior to the four markers individually.

### 2.4. Cell-Free RNA Content in Peripheral Blood Is Closely Associated with the Prognosis of NSCLC Patients

**Figure 3:**
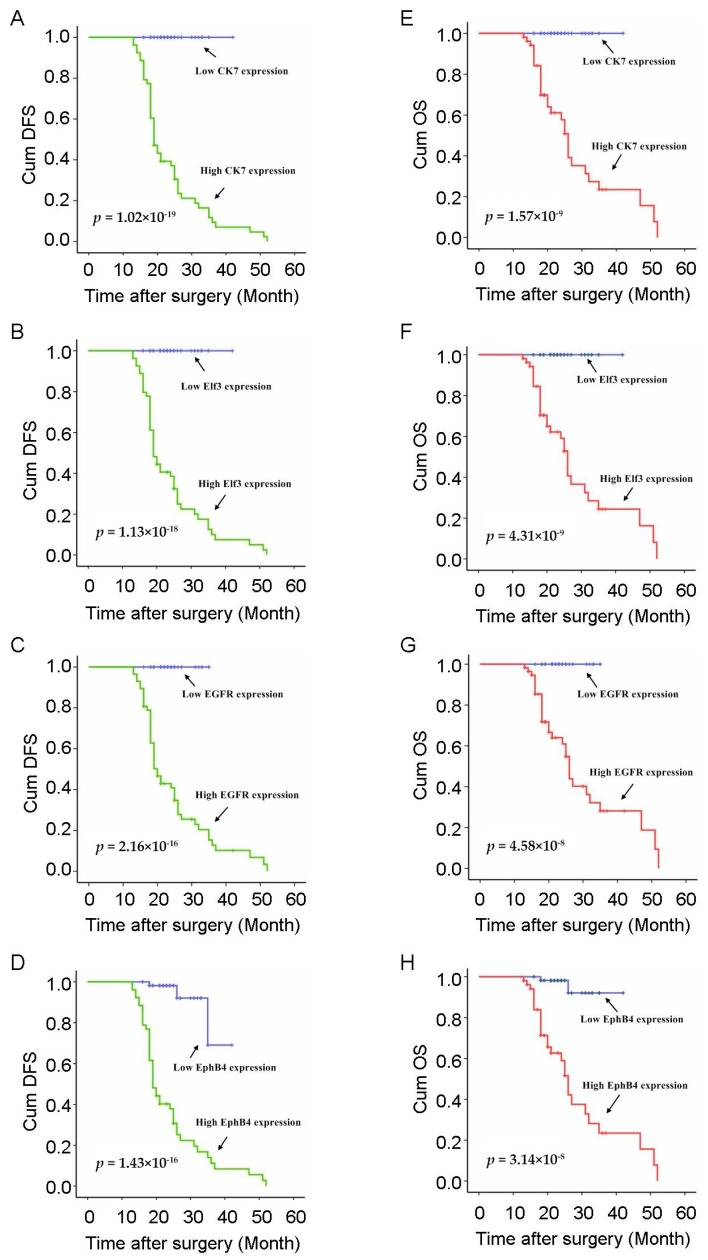
Kaplan-Meier survival analysis of DFS and OS based on the selected gene expression. Comparison of survival data from NSCLC patients positive or negative for epithelial cells in blood. Cum: Cumulative; (**A**) Kaplan-Meier curves of DFS in patients with NSCLC treated with curative surgery according to the expression of circulating *CK7*; (**B**) Kaplan-Meier curves of DFS in patients with NSCLC treated with curative surgery according to the expression of circulating *ELF3*; (**C**) Kaplan-Meier curves of DFS in patients with NSCLC treated with curative surgery according to the expression of circulating *EGFR*; (**D**) Kaplan-Meier curves of DFS in patients with NSCLC treated with curative surgery according to the expression of circulating *EphB4*; (**E**) Kaplan-Meier curves of OS in patients with NSCLC treated with curative surgery according to the expression of circulating *CK7*; (**F**) Kaplan-Meier curves of OS in patients with NSCLC treated with curative surgery according to the expression of circulating *ELF3*; (**G**) Kaplan-Meier curves of OS in patients with NSCLC treated with curative surgery according to the expression of circulating *EGFR*; (**H**) Kaplan-Meier curves of OS in patients with NSCLC treated with curative surgery according to the expression of circulating *EphB4*.

Next, we investigated the association of circulating *CK7*, *ELF3*, *EGFR*, and *EphB4* mRNA with patients’ survival. In both disease-free survival (DFS) and overall survival (OS) situations, the four circulating mRNAs were significantly associated with poor prognosis, short DFS, and low OS, respectively. The average durations of DFS and OS in patients with high level circulating *CK7*, *ELF3*, *EGFR*, and *EphB4* mRNAs (tumor PBMCs vs. positive control: >2-fold) were significantly shorter than those of patients with normal or downregulation of the four mRNAs in the PBMCs (Figure 3). The average DFS in patients with high level circulating *CK7*, *ELF3*, *EGFR*, and *EphB4* mRNAs was 17.6, 17.9, 18.4, and 17.7 months vs. 24.3, 24.2, 24.0, and 24.1 months in patients with a normal or low level of the four mRNAs, respectively. The average OS in patients with high level circulating *CK7*, *ELF3*, *EGFR*, and *EphB4* mRNA was 22.7, 22.9, 23.1, 22.6 months vs. 24.3, 24.2, 24.0, and 24.4 months in patients with a normal or low level of the four mRNAs, respectively (Table 3). All of the four circulating mRNAs, including *CK7*, *ELF3*, *EGFR*, and *EphB4* mRNA, were strongly associated with NSCLC patients’ prognosis; however, the association of the cell-free RNA content with patient survival was independent of the tumor stage (Figure 3). In addition to the four circulating mRNAs, we observed that a number of other previously characterized clinical parameters were associated with patient survival (Table 3), including gender, smoking, and TNM stage. However, no significant correlation was found between a majority of the clinical characteristics such as age at diagnosis, lesion site, histopathological type, tumor size, differentiation, invasive depth, nodal metastasis, and distant metastasis with the patients’ prognosis. In summary, these data indicate that in addition to a minority of clinical characteristics that have been shown to affect the prognosis of NSCLC patients, these four circulating mRNAs might serve as a biomarker to predict the DFS and OS of the patients.

## 3. Discussion

Metastasis is a multistep event. Some tumor cells acquire metastasis ability independent of the in situ tumor and then migrate or invade into the blood and lymph vessels to act as circulating tumor cells [15]. More than 90% of the tumor-related deaths arise out of the progression of hematogenously disseminated metastasis [16]. CTCs play a vital role in metastasis, and detection of CTCs can contribute towards the evaluation of progression, prognosis, and personal treatment [4,5]. However, because of low quantity and heterogeneity of CTCS, a unified detection method to count and classify CTCs has not yet been described [5,6]. Herein, we collected PBMCs from 111 NSCLC patients to detect the target genes’ expression originating from epithelium instead of acquiring and examining the CTCs. The elevated levels of gene expression from epithelial origin indicate the existence of CTCs or circulating tumor nuclei in peripheral blood; these cannot be detected in the normal peripheral blood [17,18]. Lung cancer is a heterogeneous disease with respect to histological and biological characteristics [19]. Owing to the heterogeneity of the tumor cells and the limited reliability of single marker, we selected four genes of epithelial origin, described previously, for the analysis of gene expression [17,18]. These four markers cannot be detected in normal mononuclear cells by optimized experiments and are specific for the detection of circulating epithelial cells [12]. The analysis of *CK7*, *ELF3*, *EGFR*, and *EphB4* mRNAs in PBMCs for the detection of CTCs have been reported in various epithelial cancers including NSCLC [11,12,13,14], respectively; however, a combined of these four markers in NSCLC has not yet been reported.

*CK7*, a star member in lung cancer, has been reported in primary or metastatic lung cancer, cavity liquid, and pleural mesothelioma. The frequency of *CK7* upregulation in adenocarcinoma (ADC) was higher than that in squamous cell carcinoma (SCC); it can be used as a potential marker to distinguish the two histological types [19,20]. However, our results were inconsistent with the previous reports. We found that neither *CK7* expression in tissues nor peripheral mononuclear cells was associated with histological types (all *p* > 0.05). *CK7* mRNA was up-regulated in all 111 NSCLC patients, including 65 ADC and 46 SCC patients. The *CK7* level in both tissues (*p* ˂ 0.0001) and PBMCs (*p* = 0.006) was significantly correlated with the clinical stage. Moreover, circulating *CK7* mRNA was significantly correlated with age during diagnosis (*p* = 0.038). NSCLC patients with low *CK7* expression (fold < 2) have a long DFS while 92.5% (49/53) patients with high *CK7* expression (fold ≥ 2) suffer progression, metastasis, or death. Therefore, *CK7* overexpression in peripheral cells may be a good biomarker for predicting poor prognosis in NSCLC. *CK7* is almost exclusively expressed in epithelial tissues, especially lung tissues. The expression of *CK7* in lung cancer tissues is elevated and can be used to distinguish between primary lung cancer and lung metastasis of other cancers [21,22,23].

*ELF3* is limited to cells of epithelial origin and is firstly found in lung tissues. Moreover, ELF3 is a transcriptional factor in the ETS-domain family and is known to regulate the expression of several growth-related genes, including angiopoietin 1, collagenase, as well as other transformed growth and invasion-related genes [24,25]. *ELF3* may play a crucial role in lung tumorigenesis and progression. However, the additional functions of *ELF3* in various kinds of tumors have frequently been studied for many years since its expression in lung cancer tissues and lung cancer cell lines was first discovered in 1997 [25]. *ELF3* was demonstrated to possess dual functions in the transcriptional regulation of genes involved in squamous epithelial differentiation. ELF3 suppresses the basal keratin4 promoter activity while simultaneously activating the late differentiation linked small proline-rich protein 2A (*SPRR2A*) promoter in both esophageal and cervical epithelial cancer cell lines [26]. Nakarai et al. evaluated the mRNA expression of *ELF3* and carcinoembryonic antigen (*CEA*) in the lymph node and the tissue from patients with colorectal cancer (CRC) and controls. The results showed that *ELF3* may sufficiently assess the lymph node metastases of CRC [27]. In addition, *ELF3* expression plays a critical role in lung tumorigenesis and is regulated by oncogenic protein kinase C (*PKC*) [28]. Our data indicate that *ELF3* expression in NSCLC tissues is associated with lymph node metastasis and clinical stage; however, its expression in PBMCs is not correlated with lymph node metastasis. Moreover, the increased *ELF3* expression correlated with poor prognosis. Hitherto, there is no report describing the association of *ELF3* expression and metastasis of lung cancer. Hence, these results should be verified by a large sample analysis in the future studies.

*EGFR* is a biomarker used for the prediction of chemotherapy and targeted treatment. It is a representative marker and abnormal expression of *EGFR* influences the treatment and prognosis of lung cancer. When patients with lung cancer carry activating *EGFR* mutations, their first-line of treatment can be selected with EGFR-TKI, like gefitinib [29]. The prognosis of NSCLC patients with mutated *EGFR* was superior to that of the wild-type *EGFR* in NSCLC [30]. We found that high *EGFR* expression level was associated with short DFS and is an independent influencing factor for the prognosis in NSCLC (*p* ˂ 0.0001). Also, it has been demonstrated that patients showed higher *EGFR* expression in SCC than non-SCC (*p* < 0.05) [31], and co-expression with insulin-like growth factor 1 receptor (*IGF1R*) was associated with poor survival. We found the *EGFR* expression did not correlate with the histological type (*p* > 0.05).

*EphB4* plays a key role in numerous kinds of tumor, including lung cancer [32], esophageal cancer [33], pancreatic cancer [34], and gliomas [35]. As a member of receptor tyrosine kinases, *EphB4* is frequently implicated in tumor pathogenesis. Moreover, the inhibition of some tyrosine kinases targets EphB4, and is deemed efficient treatment Ferguson et al. [36] found that *EphB4* overexpression promotes cellular proliferation, colony formation, and motility while *EphB4* inhibition reduces cell viability in vitro. The growth of the established tumors in mouse xenograft models can be halted by single-target strategy, and EphB4 may be a potential novel therapeutic target in lung cancer. *EphB4* expression in tumors was increased significantly compared to the control (adjacent normal tissues), which was correlated with differentiation, lymph node metastasis, and TNM stage [32]. However, we found that *EphB4* expression in PBMCs was not associated with the differentiation and lymph node metastasis, but correlated with the histological type and TNM stage. The survival curve of EphB4 was different from the other three genes. The patients with low *EphB4* expression also underwent progression, metastasis, or death.

## 4. Material and Methods

### 4.1. Patients

A total of 111 NSCLC patients who underwent curative surgery, without prior treatments, at Zhejiang Cancer Hospital (Hangzhou, China) from January 2010 to December 2011, were enrolled in this study. All the patients showed no history of other tumors by examination of a plain chest radiograph, CT scan, fiber optic bronchoscopy, and bone scan and were diagnosed with NSCLC by histopathology. The patients’ medical records were reviewed to obtain data including age at diagnosis, sex, and smoking history (Table 1). Tumor stage was determined according to the American Joint Committee on Cancer (AJCC)/ Union for International Cancer Control (UICC) TNM tumor classification [37]. The mean age of the patients at tumor resection was 62 years (range 44–82 years); 79 (71.2%) were males and 32 (28.8%) were females. The tumor specimens and paired PCHNTs were collected at the time of surgery. The paired PCHNTs were obtained from the proximity of 5 cm from the tumor edge and was assessed microscopically for the presence of healthy cells and absence of dysplastic cells. The paired peripheral blood samples were collected from these patients during diagnosis before surgery, radiation, or chemotherapy. The tissue and blood specimens for the present study were procured and used after obtaining informed consent from all the participants. As a measure of prognosis, we analyzed the clinical data concerning DFS and OS. All recruited patients had been followed-up periodically until the due date. The mean follow-up duration was 29 months; the average survival time was 32 months (range, 13–52 months) for all patients.

In addition, peripheral blood mononuclear cells (PBMCs) from 115 benign pulmonary disease patients and 120 healthy subjects as normal controls were also included to examine the specificity of these markers. PBMCs were prepared as described below.

Informed consent was obtained from all individual participants included in the study. The research involved human participants. The study was approved by the Zhejiang Cancer Hospital Institutional Review Board (2012KYB035).

### 4.2. Sample Collection and Processing

Biopsies of NSCLC and paired PCHNTs were obtained from thoracic surgery patients. The peripheral blood samples were obtained from NSCLC patients. Peripheral blood samples from 115 benign pulmonary disease patients and 120 healthy subjects were provided by our hospital. Fresh tissue samples (<300 mg) were immediately ground into powder in liquid nitrogen. Total RNA was extracted from these samples using 1 mL TRIzol (Invitrogen, Carlsbad, CA, USA) and stored at −80 °C until further use. The peripheral blood samples were collected and processed as described previously [37]. In order to avoid contamination with skin epithelial cells, the first 2 mL blood was discarded while collecting the blood samples (7.5 mL). The PBMCs containing CTCs were prepared using Red Blood Cell Lysis Buffer (Solarbio, Beijing, China) and the blood samples were processed within 30 min. After centrifugation at 2000× *g* for 15 min, buffy coat was collected and washed once with phosphate buffered saline (PBS; containing 0.14 g/L KH_2_PO_4_, 9 g/L NaCl, 0.8 g/L Na_2_HPO_4_, pH 7.4). The cells were pelleted by centrifugation. Subsequently, the tumor cell enrichment using anti-EpCAM antibodies, MACS HEA MicroBeads^®^, density gradient centrifugation, and OncoQuick^®^ plus (Miltenyi Biotec Inc.,Bergisch Gladbach, Germany) was performed as described previously [17,38,39,40]. For each cytometric enrichment procedure, 15 mL peripheral blood was used.

### 4.3. RNA Extraction and cDNA Synthesis

Total RNA of fresh tissue was isolated with TRIzol (Invitrogen, Carlsbad, CA, USA). The total RNA of PBMCs was extracted using the MiRNeasy Mini Kit (Qiagen, Hilden, Germany), according to the manufacturer’s instructions. The concentration and purity of the total RNAs were assessed by Ultraviolet spectrophotometer, and the integrity of total RNAs was estimated by polyacrylamide electrophoresis (PAGE). cDNA synthesis was performed using PrimeScript^TM^ RT reagent Kit (Takara, Otsu, Japan) for tissue RNA and PrimeScript^TM^ miRNA cDNA Synthesis Kit (Takara, Otsu, Japan) for PBMCs RNA.

### 4.4. Real-Time RT-PCR

The mRNA levels of the genes of interest were estimated by real-time RT-PCR on Applied Biosystems 7500 (Foster City, CA, USA) using SYBR^®^ Premix Ex Taq^TM^ II (Takara, Otsu, Japan). The sequences of primers were designed as described previously [17,18]. To prepare an artificial CTC model as a positive control, a trace amount of A549 human lung adenocarcinoma cells was mixed with peripheral blood samples (five A549 cells in 7.5 mL peripheral blood). Then, the total RNA was extracted from the mixture and used as the positive control in real-time RT-PCR. The total amount of mRNA was normalized to *GAPDH*, and the relative mRNA expressions of the samples (tumor vs. PCHNTs, or paired PBMCs vs. positive control) were calculated by 2^−ΔΔ*C*t^. The differential expression of the samples may be defined as the upregulation when the cut-off value was set at 2-fold.

### 4.5. Statistical Analysis

The SPSS 17.0 statistical software package was used for all the statistical analyses. The correlation between the level of the above described four mRNAs and the clinicopathological characteristics was analyzed by Person’s chi-square test or Fisher’s exact test. The survival curves and univariate analysis were generated by the Kaplan-Meier method and log-rank test. Multivariate analysis was performed by the Cox regression model and Wald test. *p* < 0.05 was considered statistically significant.

## 5. Conclusions

In present study, we selected *CK7*, *ELF3*, *EGFR* and *EphB4*, four epithelial origin markers, to evaluate their efficiency for detecting CTCs in NSCLC. The expression of *CK7*, *ELF3*, *EGFR* and *EphB4* in NSCLC tissues and para PMBCs were both upregulated significantly. ROC curve analysis indicated that these four genes have high sensitivity and specificity for detecting CTCs. Moreover, upregulation of four genes in PMBCs are associated with tumor progression closely. The patients with high expression of these genes in PMBCs have poor survival. We speculated that *CK7*, *ELF3*, *EGFR* and *EphB4* expression in PMBCs are appropriate markers for detecting CTCs and have potential in the evaluation of prognosis and monitoring of therapy in NSCLC.

## Figures and Tables

**Table 1:** Correlation of *CK7*, *ELF3*, *EGFR*, *EphB4* expression in tissues with NSCLC’s clinicopathological characteristics.

Clinicopathological Parameters		*N*	CK7		ELF3		EGFR		EphB4	
			Mean ± SD	*p*-Value	Mean ± SD	*p*-Value	Mean ± SD	*p*-Value	Mean ± SD	*p*-Value
Gender	Male	79	36.73 ± 19.44	0.608	36.64 ± 19.60	0.330	35.23 ± 20.76	0.590	32.98 ± 19.65	0.571
	Female	32	34.60 ± 29.66		32.59 ± 20.20		32.84 ± 21.87		30.64 ± 19.76	
Age (year)	<60	46	36.54 ± 18.05	0.852	36.24 ± 18.25	0.728	36.58 ± 20.62	0.393	34.41 ± 19.20	0.345
	≥60	65	35.82 ± 20.96		34.93 ± 20.89		33.10 ± 21.33		30.82 ± 19.92	
Smoking history	No	46	36.27 ± 21.05	0.947	34.45 ± 20.20	0.646	34.08 ± 20.61	0.847	32.84 ± 19.78	0.812
	Yes	65	36.01 ± 18.90		36.21 ± 19.57		34.87 ± 21.45		31.93 ± 19.65	
Lesion site	Left lobe	50	34.45 ± 17.73	0.413	34.92 ± 19.38	0.790	33.65 ± 20.14	0.686	31.78 ± 18.85	0.797
	Right lobe	61	37.49 ± 21.27		35.93 ± 20.22		35.28 ± 21.85		32.74 ± 20.37	
Histopathological type	SCC	46	34.46 ± 18.00	0.460	35.14 ± 19.34	0.879	32.67 ± 19.35	0.423	31.03 ± 19.23	0.567
	ADC	65	37.29 ± 20.92		35.72 ± 20.21		35.86 ± 22.17		33.21 ± 19.99	
Tumor size	≤3 cm	24	37.47 ± 22.02	0.727	35.88 ± 20.95	0.938	36.82 ± 24.34	0.615	30.90 ± 18.19	0.653
	>3 cm	86	35.86 ± 19.25		35.52 ± 19.61		34.07 ± 20.18		32.94 ± 20.08	
Differentiation	Well/Moderate	86	34.84 ± 19.72	0.207	34.09 ± 19.47	0.173	33.23 ± 21.31	0.224	30.56 ± 19.60	0.082
	Poor	25	40.51 ± 19.51		40.23 ± 20.42		39.05 ± 19.73		38.32 ± 18.81	
T stage	T2	94	35.55 ± 19.70	0.476	34.63 ± 19.68	0.289	33.33 ± 20.89	0.153	30.78 ± 19.18	0.053
	T3	17	39.27 ± 20.16		40.18 ± 20.14		41.27 ± 21.08		40.77 ± 20.45	
Lymph node status	N0	18	31.75 ± 13.75	0.188	30.02 ± 13.90	0.105	30.47 ± 15.85	0.275	28.47 ± 15.94	0.296
	N1–3	93	36.96 ± 20.64		36.53 ± 20.60		35.33 ± 21.86		33.05 ± 20.24	
Distant metastasis	M0	109	36.20 ± 19.85	0.735	35.56 ± 19.87	0.748	34.64 ± 21.12	0.721	32.39 ± 19.67	0.745
	M1	2	31.40 ± 14.85		31.00 ± 16.69		29.25 ± 18.31		27.80 ± 22.49	
Clinical stage	I/II	56	28.13 ± 12.80	**1.06 × 10^−5^**	27.09 ± 13.95	**3.23 × 10^−6^**	25.14 ± 15.18	**7.62 × 10^−7^**	23.87 ± 14.95	**2.05 × 10^−6^**
	III/IV	55	44.25 ± 22.18		44.01 ± 21.22		44.11 ± 21.91		40.90 ± 20.18	

SCC: squamous cell carcinoma; ADC: adenocarcinoma; Mean: mean value of relative expression; SD: standard deviation; Numbers in bold: *p* < 0.05.

**Table 2:** Correlation of *CK7*, *ELF3*, *EGFR*, *EphB4* expression in PBMCs with NSCLC’s clinicopathological characteristics.

Clinicopathological Parameters		*N*	CK7		ELF3		EGFR		EphB4	
			Up-Regulated (%)	χ2	Up-Regulated (%)	χ2	Up-Regulated (%)	χ2	Up-Regulated (%)	χ2
				*p*-Value		*p*-Value		*p*-Value		*p*-Value
Gender	Male	79	40 (50.63)	0.432	41 (51.89)	0.605	41 (51.90)	0.014	39 (49.37)	0.699
	Female	32	14 (43.75)	0.511	14 (43.75)	0.437	17 (55.13)	0.907	13 (40.63)	0.403
Age (year)	<60	46	17 (36.96)	4.299	20 (43.47)	1.158	22 (47.83)	0.617	19 (41.30)	0.969
	≥60	65	37 (56.92)	**0.038**	35 (53.84)	0.282	36 (55.38)	0.432	33 (50.77)	0.325
Smoking history	No	46	26 (56.52)	1.949	26 (56.52)	1.528	28 (60.87)	2.338	24 (52.17)	0.895
	Yes	65	28 (43.08)	0.163	29 (44.61)	0.216	30 (46.15)	0.126	28 (43.08)	0.344
Lesion site	Left lobe	50	25 (50.00)	0.285	24 (48.00)	0.000	26 (52.00)	0.055	21 (42.00)	0.437
	Right lobe	61	28 (45.90)	0.593	30 (49.18)	0.983	31 (50.82)	0.815	30 (49.18)	0.509
Histopathological type	SCC	46	19 (41.30)	1.696	20 (43.47)	1.158	22 (47.83)	0.617	16 (34.78)	4.592
	ADC	65	35 (53.85)	0.193	35 (53.84)	0.282	36 (55.38)	0.432	36 (55.38)	**0.032**
Tumor size	≤3 cm	24	13 (54.17)	0.316	14 (58.33)	0.853	14 (58.33)	0.387	15 (62.50)	2.856
	>3 cm	86	41 (47.67)	0.574	41 (47.67)	0.356	44 (51.16)	0.534	37 (43.02)	0.091
Differentiation	Well/Moderate	86	43 (50.00)	0.279	42 (48.83)	0.078	44 (51.16)	0.182	39 (45.35)	0.344
	Poor	25	11 (44.00)	0.597	13 (52.00)	0.781	14 (56.00)	0.670	13 (52.00)	0.557
T stage	T2	94	46 (48.94)	0.020	46 (48.93)	0.092	47 (50.00)	1.248	43 (45.74)	0.299
	T3	17	8 (47.06)	0.887	9 (52.94)	0.761	11 (64.71)	0.264	9 (52.94)	0.584
Lymph node metastasis	N0	18	5 (27.78)	3.746	7 (38.88)	0.977	5 (27.78)	5.158	6 (33.33)	1.576
	N1–3	93	48 (52.17)	0.053	47 (51.08)	0.323	52 (56.52)	**0.023**	45 (48.95)	0.209
Distant metastasis	M0	109	53 (48.62)	0.000	54 (49.54)	0.000	57 (52.29)	0.004	51 (46.79)	0.000
	M1	2	1 (50.00)	1.000	1 (50.00)	1.000	1 (50.00)	0.949	1 (50.00)	1.000
Clinical stage	I/II	56	20 (35.71)	7.569	20 (35.71)	8.654	19 (33.93)	15.210	19 (33.93)	7.574
	III/IV	55	34 (61.82)	**0.006**	35 (63.63)	**0.003**	39 (70.91)	**9.62 × 10^−5^**	33 (60.00)	**0.006**

SCC: squamous cell carcinoma; ADC: adenocarcinoma; up-regulated: the number of patients with gene upregulation; Numbers in bold: *p* < 0.05.

**Table 3:** Univariate and multivariate analysis of survival in 110 patients with NSCLC according to clinicopathologic factors and circulating mRNA levels of *CK7*, *ELF3*, *EGFR* and *EphB4.*

Clinicopathologic Factor		DFS							OS					
		Total	Survival (mo.)	Univariate Analysis		Multivariate Analysis			Survival (mo.)	Univariate Analysis		Multivariate Analysis		
		*N*		χ2	*p*-Values	HR (95% CI)		*p*-Values		χ2	*p*-Values	HR (95% CI)		*p*-Values
Age	<60	45	21.69 ± 6.90	2.577	0.108	1.604	0.842–3.057	0.151	22.67 ± 6.43	2.608	0.106	2.184	0.894–5.337	0.086
	≥60	65	20.66 ± 7.75						24.15 ± 7.76					
Gender	Male	78	19.92 ± 6.85	4.447	0.035	0.237	0.098–0.574	0.001	22.65 ± 5.95	8.597	0.003	0.120	0.026–0.553	0.006
	Female	32	23.91 ± 8.02						25.72 ± 9.50					
Smoking	No	45	21.33 ± 7.95	0.580	0.446	0.456	0.215–0.969	0.041	23.96 ± 8.00	0.707	0.401	0.578	0.220–1.519	0.266
	Yes	65	20.91 ± 7.05						23.26 ± 6.74					
Lesion site	Left lobe	50	20.94 ± 7.79	0.003	0.957	0.873	0.476–1.601	0.660	24.26 ± 7.74	0.254	0.615	0.933	0.409–2.130	0.869
	Right lobe	60	21.20 ± 7.12						22.95 ± 6.83					
Histopathological type	SCC	45	22.60 ± 7.85	2.286	0.131	1.374	0.654–2.888	0.401	24.76 ± 7.76	0.068	0.794	1.338	0.518–3.452	0.548
	ADC	65	20.03 ± 6.94						22.71 ± 6.82					
Tumor size	≤3 cm	24	20.38 ± 6.28	0.833	0.361	0.894	0.421–1.897	0.769	24.12 ± 8.22	0.342	0.559	1.528	0.476–4.903	0.476
	>3 cm	85	21.34 ± 7.73						23.47 ± 7.00					
Differentiation	Well/Moderate	40	21.55 ± 6.92	0.016	0.898	0.685	0.326–1.439	0.318	23.80 ± 6.43	0.000	1.000	0.653	0.237–1.796	0.409
	Poor	70	20.81 ± 7.70						23.40 ± 7.73					
T stage	T2	93	20.92 ± 7.23	0.000	0.993	0.776	0.313–1.925	0.584	23.30 ± 6.92	0.269	0.604	0.410	0.120–1.407	0.156
	T3	17	21.94 ± 8.45						24.88 ± 8.99					
Lymph node status	N0	18	19.28 ± 4.99	0.585	0.444	0.757	0.256–2.243	0.616	20.39 ± 3.27	0.455	0.500	0.497	0.133–1.859	0.299
	N1–3	92	21.43 ± 7.76						24.16 ± 7.66					
Distant metastasis	M0	108	20.82 ± 7.09	2.229	0.135	0.000	0.000–9.74 × 10^233^	0.968	23.28 ± 6.82	0.417	0.519	1.451	0.139–15.122	0.756
	M1	2	35.00 ± 14.14						38.00 ± 18.38					
Clinical stage	I/II	56	21.96 ± 6.89	5.723	0.017	1.878	0.903–3.903	0.091	23.29 ± 6.58	1.075	0.300	1.418	0.532–3.781	0.486
	III/IV	54	20.17 ± 7.85						23.81 ± 7.95					
Circulating CK7 mRNA	>2-fold	53	17.62 ± 7.95	82.565	1.02 × 10^−9^	132.315	10.691-1637.622	1.41 × 10^−4^	22.74 ± 9.00	36.447	1.57 × 10^−9^	91.148	3.863–2150.588	0.005
	Normal/low	57	24.30 ± 5.11						24.30 ± 5.11					
Circulating ELF3 mRNA	>2-fold	54	17.87 ± 8.00	77.808	1.13 × 10^−18^	119.681	10.024–1428.965	1.56 × 10^−4^	22.89 ± 8.91	34.479	4.31 × 10^−9^	84.458	3.655–1951.764	0.006
	Normal/low	56	24.18 ± 5.19						24.18 ± 5.19					
Circulating EGFR mRNA	>2-fold	57	18.39 ± 8.51	67.452	2.16 × 10^−16^	101.954	8.565–1213.683	2.53 × 10^−4^	23.14 ± 9.13	29.888	4.58 × 10^−8^	72.525	3.178–1655.090	0.007
	Normal/low	53	23.98 ± 4.49						23.98 ± 4.49					
Circulating EphB4 mRNA	>2-fold	52	17.69 ± 7.69	68.262	1.43 × 10^−16^	26.490	8.111–86.516	5.76 × 10^−8^	22.62 ± 8.88	30.619	3.14 × 10^−8^	19.010	4.487–80.547	6.40 × 10^−5^
	Normal/low	58	24.12 ± 5.64						24.38 ± 5.34					

SCC: squamous cell carcinoma; ADC: adenocarcinoma; DFS: disease free survival; OS: overall survival; HR: hazard ratio; CI: confidence interval; mo.: month; Normal: 0.5 ≤ 2^−ΔΔ*C*^^t^ ≤ 2; low: 2^−ΔΔ*C*^^t^ < 0.5.

## References

[1] Kalia M. (2015). Biomarkers for personalized oncology: Recent advances and future challenges. Metabolism.

[2] Okimoto R.A., Bivona T.G. (2014). Recent advances in personalized lung cancer medicine. Per. Med..

[3] Kathuria H., Gesthalter Y., Spira A., Brody J.S., Steiling K. (2014). Updates and controversies in the rapidly evolving field of lung cancer screening, early detection, and chemoprevention. Cancers.

[4] Ilie M., Hofman V., Long E., Bordone O., Selva E., Washetine K., Marquette C.H., Hofman P. (2014). Current challenges for detection of circulating tumor cells and cell-free circulating nucleic acids, and their characterization in non-small cell lung carcinoma patients. What is the best blood substrate for personalized medicine?. Ann. Transl. Med..

[5] Diaz L.A., Bardelli A. (2014). Liquid biopsies: Genotyping circulating tumor DNA. J. Clin. Oncol..

[6] Joosse S.A., Gorges T.M., Pantel K. (2014). Biology, detection, and clinical implications of circulating tumor cells. EMBO Mol. Med..

[7] Han Y., Su C., Liu Z. (2014). Methods for detection of circulating cells in non-small cell lung cancer. Front. Biosci..

[8] Warkiani M.E., Khoo B.L., Tan D.S., Bhagat A.A., Lim W.T., Yap Y.S., Lee S.C., Soo R.A., Han J., Lim C.T. (2014). An ultra-high-throughput spiral microfluidic biochip for the enrichment of circulating tumor cells. Analyst.

[9] De Albuquerque A., Kubisch I., Stölzel U., Ernst D., Boese-Landgraf J., Breier G., Stamminger G., Fersis N., Kaul S. (2012). Prognostic and predictive value of circulating tumor cell analysis in colorectal cancer patients. J. Transl. Med..

[10] Li Q., Qi H., Zhou H.X., Deng C.Y., Zhu H., Li J.F., Wang X.L., Li F.R. (2011). Detection of micrometastases in peripheral blood of non-small cell lung cancer with a refined immunomagnetic nanoparticle enrichment assay. Int. J. Nanomed..

[11] Felton T., Harris G.C., Pinder S.E., Snead D.R., Carter G.I., Bell J.A., Haines A., Kollias J., Robertson J.F., Elston C.W. (2004). Identification of carcinoma cells in peripheral blood samples of patients with advanced breast carcinoma using RT-PCR amplification of CK7 and MUC1. Breast.

[12] Raynor M., Stephenson S.A., Walsh D.C., Pittman K.B., Dobrovic A. (2002). Optimisation of the RT-PCR detection of immunomagnetically enriched carcinoma cells. BMC Cancer.

[13] Zhang X., Xie J., Yu C., Yan L., Yang Z. (2014). mRNA expression of CK19, EGFR and LUNX in patients with lung cancer micrometastasis. Exp. Ther. Med..

[14] Tang X.X., Brodeur G.M., Campling B.G., Ikegaki N. (1999). Coexpression of transcripts encoding EPHB receptor protein tyrosine kinases and their ephrin-B ligands in human small cell lung carcinoma. Clin. Cancer Res..

[15] Chiang A.C., Massagué J. (2008). Molecular basis of metastasis. N. Engl. J. Med..

[16] Wicha M.S., Hayes D.F. (2011). Circulating tumor cells: Not all detected cells are bad and not all bad cells are detected. J. Clin. Oncol..

[17] Man Y., Cao J., Jin S., Xu G., Pan B., Shang L., Che D., Yu Q., Yu Y. (2014). Newly identified biomarkers for detecting circulating tumor cells in lung adenocarcinoma. Tohoku J. Exp. Med..

[18] Winter S.C., Stephenson S.A., Subramaniam S.K., Paleri V., Ha K., Marnane C., Krishnan S., Rees G. (2009). Long term survival following the detection of circulating tumor cells in head and neck squamous cell carcinoma. BMC Cancer.

[19] Cheng X., Chen H. (2014). Tumor heterogeneity and resistance to EGFR-targeted therapy in advanced nonsmall cell lung cancer: Challenges and perspectives. Onco Targets Ther..

[20] Camilo R., Capelozzi V.L., Siqueira S.A.C., Bernardi F.D.C. (2006). Expression of p63, keratin 5/6, keratin 7, and surfactant-A in non-small cell lung carcinomas. Hum. Pathol..

[21] Ikeda S., Fujimori M., Shibata S., Okajima M., Ishizaki Y., Kurihara T., Miyata Y., Iseki M., Shimizu Y., Tokumoto N. (2006). Combined immunohistochemistry of β-catenin, cytokeratin 7, and cytokeratin 20 is useful in discriminating primary lung adenocarcinomas from metastatic colorectal cancer. BMC Cancer.

[22] Chhieng D.C., Cangiarella J.F., Zakowski M.F., Goswami S., Cohen J.M., Yee H.T. (2001). Use of thyroid transcription factor 1, PE-10, and cytokeratins 7 and 20 in discriminating between primary lung carcinomas and metastatic lesions in fine-needle aspiration biopsy specimens. Cancer.

[23] Gruver A.M., Amin M.B., Luthringer D.J., Westfall D., Arora K., Farver C.F., Osunkoya A.O., McKenney J.K., Hansel D.E. (2012). Selective immunohistochemical markers to distinguish between metastatic high-grade urothelial carcinoma and primary poorly differentiated invasive squamous cell carcinoma of the lung. Arch. Pathol. Lab. Med..

[24] Thomas R.S., Ng A.N., Zhou J., Tymms M.J., Doppler W., Kola I. (2000). The Elf group of Ets-related transcription factors. ELF3 and ELF5. Adv. Exp. Med. Biol..

[25] Tymms M.J., Ng A.Y., Thomas R.S., Schutte B.C., Zhou J., Eyre H.J., Sutherland G.R., Seth A., Rosenberg M., Papas T. (1997). A novel epithelial-expressed ETS gene, *ELF3*: Human and murine cDNA sequences, murine genomic organization, human mapping to 1q32.2 and expression in tissues and cancer. Oncogene.

[26] Brembeck F.H., Opitz O.G., Libermann T.A., Rustgi A.K. (2000). Dual function of the epithelial specific ets transcription factor, ELF3, in modulating differentiation. Oncogene.

[27] Nakarai C., Osawa K., Matsubara N., Ikeuchi H., Yamano T., Okamura S., Kamoshida S., Tsutou A., Takahashi J., Ejiri K. (2012). Significance of ELF3 mRNA expression for detection of lymph node metastases of colorectal cancer. Anticancer Res..

[28] Erdogan E., Klee E.W., Thompson E.A., Fields A.P. (2009). Meta-analysis of oncogenic protein kinase Ciota signaling in lung adenocarcinoma. Clin. Cancer Res..

[29] Mok T.S., Wu Y.L., Thongprasert S., Yang C.H., Chu D.T., Saijo N., Sunpaweravong P., Han B., Margono B., Ichinose Y. (2009). Gefitinib or carboplatin-paclitaxel in pulmonary adenocarcinoma. N. Engl. J. Med..

[30] Liang W., Zhang Y., Kang S., Pan H., Shao W., Deng Q., Shi X., Wang W., He J. (2014). Impact of EGFR mutation status on tumor response and progression free survival after first-line chemotherapy in patients with advanced non-small-cell lung cancer: A meta-analysis. J. Thorac. Dis..

[31] Gately K., Forde L., Cuffe S., Cummins R., Kay E.W., Feuerhake F., O’Byrne K.J. (2014). High coexpression of both EGFR and IGF1R correlates with poor patient prognosis in resected non-small-cell lung cancer. Clin. Lung Cancer.

[32] Zheng M.F., Ji Y., Wu X.B., Ye S.G., Chen J.Y. (2012). *EphB4* gene polymorphism and protein expression in non-small-cell lung cancer. Mol. Med. Rep..

[33] Hasina R., Mollberg N., Kawada I., Mutreja K., Kanade G., Yala S., Surati M., Liu R., Li X., Zhou Y. (2013). Critical role for the receptor tyrosine kinase EPHB4 in esophageal cancers. Cancer Res..

[34] Li M., Zhao J., Qiao J., Song C., Zhao Z. (2014). EphB4 regulates the growth and migration of pancreatic cancer cells. Tumour Biol..

[35] Chen T., Liu X., Yi S., Zhang J., Ge J., Liu Z. (2013). EphB4 is overexpressed in gliomas and promotes the growth of glioma cells. Tumor Biol..

[36] Ferguson B.D., Liu R., Rolle C.E., Tan Y.H., Krasnoperov V., Kanteti R., Tretiakova M.S., Cervantes G.M., Hasina R., Hseu R.D. (2013). The EphB4 receptor tyrosine kinase promotes lung cancer growth: A potential novel therapeutic target. PLoS ONE.

[37] Edge S.B., Compton C.C. (2010). The American Joint Committee on Cancer: The 7th edition of the AJCC cancer staging manual and the future of TNM. Ann. Surg. Oncol..

[38] Hayes D.C., Secrist H., Bangur C.S., Wang T., Zhang X., Harlan D., Goodman G.E., Houghton R.L., Persing D.H., Zehentner B.K. (2006). Multigene real-time PCR detection of circulating tumor cells in peripheral blood of lung cancer patients. Anticancer Res..

[39] Xi L., Nicastri D.G., El-Hefnawy T., Hughes S.J., Luketich J.D., Godfrey T.E. (2007). Optimal markers for real-time quantitative reverse transcription PCR detection of circulating tumor cells from melanoma, breast, colon, esophageal, head and neck, and lung cancers. Clin. Chem..

[40] Königsberg R., Gneist M., Jahn-Kuch D., Pfeiler G., Hager G., Hudec M., Dittrich C., Zeillinger R. (2010). Circulating tumor cells in metastatic colorectal cancer: Efficacy and feasibility of different enrichment methods. Cancer Lett..

